# Meat consumers and non-meat consumers in Germany: a characterisation based on results of the German National Nutrition Survey II

**DOI:** 10.1017/jns.2019.17

**Published:** 2019-06-07

**Authors:** Franziska Koch, Thorsten Heuer, Carolin Krems, Erika Claupein

**Affiliations:** Department of Nutritional Behaviour, Max Rubner-Institut, Federal Research Institute of Nutrition and Food, Karlsruhe, Haid-und-Neu-Strasse 9, 76131 Karlsruhe, Germany

**Keywords:** Meat consumption, Vegetarian diet, Health, Food consumption, German National Nutrition Survey II, NVS II, German National Nutrition Survey II

## Abstract

Meat consumption in high-income countries is increasingly discussed due to its impact on environment and health as well as ethical considerations. The present paper aims to provide information on meat consumption behaviour, sociodemographic factors related to meat consumption and its associations with health and nutritional behaviour, based on the German National Nutrition Survey II. For 12 915 participants aged 18–80 years, food consumption was assessed by two 24-h recalls and further data by interviews. Participants were distinguished in non-meat consumers and meat consumers; meat consumers were further differentiated as low and high meat consumers (<86 g/d and ≥86 g/d). Group differences were analysed using binary logistic and linear regression models. More non-meat consumers were found among women, young and more educated persons. They showed equal or more preferable health characteristics, had a similar energy intake but ate more plant-based foods compared with meat consumers. More high meat consumers were found among men, young and middle-aged and lower-educated persons. Compared with low meat consumers, they showed equal or less preferable health characteristics, had a higher energy intake and ate more potatoes and sauces/spices and less of most other food groups in relation to their energy intake. To conclude, sociodemographic groups differ in their meat consumption and differences in meat consumption go together with differences in health behaviour and other food consumption.

In recent years, the high meat consumption in high-income countries has been increasingly criticised in science and society. Causes of concern are the ethical, but more recently the health and environmental implications of meat production and consumption^(^^1^^–^^3^^)^.

Ethical concerns about meat consumption focus on the killing of animals for human nutrition in general and the industrial production methods. Even many meat consumers experience difficulties as they do not want to hurt animals but like to eat meat at the same time (also called the ‘meat paradox’^(^^4^^)^). However, meat consumption is rationalised by many consumers with the 4Ns: eating meat is regarded natural, normal, necessary and nice^(^^5^^)^. In terms of health, meat is appreciated for its nutritional value as a source of high-quality protein, Fe, Zn and vitamin B_12_, but its natural content of saturated fat and cholesterol as well as some substances added in processing or formed during the cooking process are areas of concern^(^^6^^)^. The high consumption levels observed in high-income countries raise public health concerns as there is epidemiological evidence for an association of meat consumption with a higher risk for common lifestyle diseases (CVD, diabetes mellitus, some types of cancer) in affluent societies^(^^7^^)^. Further public health risks attributable to meat production and consumption include the increase in antibiotic resistance in humans and risks from pesticide residues and foodborne pathogens^(^^8^^)^ as well as the adverse health effects due to climate change^(^^9^^,^^10^^)^. High meat production and consumption are also associated with numerous environmental issues. As mentioned before, meat consumption is a key issue in discussions about climate change. Different studies suggest that about 18–50 % of global greenhouse gas emissions are attributable to livestock production^(^^11^^,^^12^^)^ – although consumers tend to underestimate this problem^(^^13^^)^. Additional environmental problems related to meat production are land degradation and deforestation, water shortage and water pollution and loss of biodiversity^(^^12^^)^.

Against this background, the majority of nutrition and health organisations recommend moderate meat consumption. The recommendation of the German Nutrition Society (DGE) is to eat not more than 300–600 g of meat per week^(^^14^^)^, which corresponds to about 43 to 86 g per d. Actually, men and women in Germany on average eat 142 and 76 g meat per d, respectively, with noticeable differences depending on age and socio-economic status^(^^15^^)^.

Given the social significance of meat consumption, it is important to provide further information on this topic, *inter alia* on consumption behaviour, the sociodemographic factors related to meat consumption and its associations with health and nutritional behaviour. The objective of this study was to use data of the German National Nutrition Survey II (NVS II) to analyse meat consumption in this regard. To explore practically relevant differences, comparisons were made (1) between persons not consuming meat and persons consuming meat in general as well as (2) between persons consuming meat in accordance with the dietary recommendations of the DGE (<86 g/d) and persons consuming more meat than recommended (≥ 86 g/d).

## Methods

### Design and subjects

The NVS II is a representative cross-sectional survey studying food consumption and further aspects of nutritional behaviour of the German population. The survey was conducted between November 2005 and January 2007 with approximately 20 000 German-speaking participants 14–80 years of age and living in private households. The NVS II is based on a two-stage sampling approach of municipalities stratified by administrative district and type (first stage) and residents based on addresses from population registries stratified by sex and age (second stage). It is constructed modularly with different dietary assessment methods applied. The NVS II was approved by the German Federal Data Protection Office. Respondents were informed in detail about the study objectives, interview and examination procedures, as well as the handling of data records and analyses under pseudonymous conditions. It was made clear that participation was on a voluntary basis and could be terminated at any time. A more detailed description of the design and assessment methods of the NVS II can be obtained from the basic result report^(^^16^^)^ and Heuer *et al*.^(^^15^^)^.

The present analysis is based on data from computer-assisted personal/telephone interviews and two 24-h recalls conducted by telephone. This dietary assessment method was chosen for reasons of comparability with future studies, especially the German National Nutrition Monitoring (NEMONIT).

As the recommendations of the German National Nutrition Society are directed towards adults, participants aged 18–80 were selected for this analysis. Due to this restriction, data weights, which were only available for the total sample, could not be applied. However, a comparison of demographic characteristics between the NVS II study sample analysed here (*n* 12 915) and the Mikrozensus (providing official representative statistics of the population in Germany) reveals no substantial deviations. Women and higher-educated persons were over-represented, which is common for nutritional surveys but needs to be reflected when interpreting the data.

### Measures

#### Food consumption and energy intake

On two non-consecutive days, 24-h recalls were conducted by telephone using the software EPIC-SOFT^(^^17^^)^ (renamed GloboDiet in 2014). All reported food items were categorised into general food groups, which were described in detail by Heuer *et al*.^(^^15^^)^. Specifically for this analysis, one new food group for soya products (e.g. soya milk, tofu) was generated and the food group ‘meat, meat products and sausages’ was further differentiated. Meats from various animals differ substantially in their environmental and health impact as well as their sociocultural meanings. Hence, all food items out of the food group meat and meat products were classified according to their origin into beef/veal/mutton/lamb, pork, poultry, special meat or meat from unknown/mixed origin (Table 1). For the food group sausages, a clear classification of the origin was not possible. If not otherwise specified, the term ‘meat’ refers to total meat including meat, meat products and sausages.

**Table 1.** Daily consumption (g) of total meat and its subgroups among meat consumers, German National Nutrition Survey II (Arithmetic means and 95 % confidence intervals)

		Men (*n* 5713)		Women (*n* 7020)		Total (*n* 12 733)	
Food group		Mean	95 % CI	Mean	95 % CI	Mean	95 % CI
Total meat (including meat, meat products and sausages)		155	152, 158	87	86, 89	118	116, 119
	Meat and meat products (unprocessed meats and meats processed for conservation and/or refinement; including offal, minced meat and meat sauces)	101	99, 103	62	60, 63	79	78, 81
	Beef, veal, mutton, lamb	14	13, 15	8	8, 9	11	10, 11
	Pork	45	44, 47	26	25, 26	34	33, 35
	Poultry (chicken, turkey, duck, goose)	18	17, 19	13	12, 14	15	15, 16
	Special meat (e.g. game, rabbit, horse, quail)	2	1, 2	1	1, 1	1	1, 1
	Unknown or mixed meat	22	21, 23	14	13, 15	18	17, 18
	Sausages (mixture of chopped meat, fat tissue and flavouring ingredients; of all origins)	54	52, 55	26	25, 27	38	38, 39

According to their meat consumption, participants were classified into one of three groups: non-meat consumers were defined as persons not consuming meat but potentially fish or seafood. Participants were classified as such if they self-identified as vegetarian (including vegan, lacto-vegetarian, ovo-vegetarian, ovo-lacto-vegetarian or pesco-ovo-lacto-vegetarian) and if they did not consume meat in both 24-h recall days (whereby small amounts of meat from standard recipes included in the software were not considered). All other participants were considered meat consumers and, according to their daily meat consumption, they were classified either as those not exceeding the maximum consumption recommended by the German Nutrition Society^(^^14^^)^ (total meat consumption <86 g/d = low meat consumers) or as those exceeding the recommended consumption (total meat consumption ≥86 g/d = high meat consumers). Both the recommendation and the classification refer to prepared/ready-to-eat meat and include all types of meat.

Energy intake (kJ/d) was calculated as the sum of energy content from all consumed foods according to the German Nutrient Database (BLS, version 3.02)^(^^18^^)^. To obtain energy density (kJ/g), energy intake (kJ/d) was divided by the amount of foods (without beverages) consumed (g/d).

### Sociodemographic and health data

Information on sociodemographic and health data was collected in a computer-assisted personal or telephone interview. Information on sex, age, country of birth and household size as well as smoking status and subjective health were directly obtained from the interview. For social status, three indicators were constructed and analysed separately: The education score was based on the educational, vocational and academic qualifications of the respondent. The score for the occupational status of the principal earner of the household was based on the type of employment and the qualification level of that person. Per capita income was calculated as monthly household net income (mid-point of class interval) divided by the number of persons in the household. For all three indicators, participants were divided into three groups of about equal size (low, middle, high). To obtain information about regional structure, municipalities were categorised according to their ‘use density’ (number of inhabitants and employees in the area) into large cities, medium cities and suburbs and small cities and rural areas^(^^19^^)^. BMI was calculated as body weight (kg) divided by body height (m) squared and categorised into underweight, normal weight, preobese and obese according to the cut-off points provided by the WHO^(^^20^^)^. Body height and weight were either measured in the personal interview or obtained from self-reports in the telephone interview. Missing values for BMI (*n* 32) were replaced by mean values calculated for sex and age. Information on sports activities (yes/no) was collected in an additional questionnaire, which was not completed by all participants in the study sample. Therefore, the analyses on sports activities are based on a smaller sample (*n* 8831).

### Statistical analysis

All statistics were performed using SAS 9.3 (SAS Institute, Inc.). As sex has been shown to be a major factor affecting meat consumption, all analyses were done separately for men and women. Categorical and metric variables are presented as percentages and arithmetic means with 95 % CI, respectively. Although values for meat consumption were not normally distributed, arithmetic means rather than median values were chosen. This presentation was used because most meat subgroups show median intakes of zero, which are of restricted use for comparisons and therefore not considered appropriate.

Prevalences of non-meat consumption, and low and high meat consumption in sociodemographic groups were compared using 95 % CI with non-overlapping intervals indicating significant differences. Independent associations between sociodemographic characteristics and meat consumption level were examined in binary logistic regression models with non-meat consumption (reference: meat consumption) and high meat consumption (reference: low meat consumption) as dependent variables. Results are presented as OR with 95 % CI, where intervals not including the value ‘1’ indicate significant differences.

Differences in health and nutritional behaviour between non-meat consumers and meat consumers as well as between high meat and low meat consumers (independent variables) were examined either using binary logistic regression models (dependent variables: health-related characteristics) or linear regression models (dependent variables: energy and food intake) as appropriate. Results are presented as unadjusted and adjusted OR (with 95 % CI) and regression coefficients (with 95 % CI, where intervals not including the value ‘0’ indicate significant differences), respectively. Adjustment was made for sociodemographic factors and/or energy intake.

To examine if categorisation into groups according to DGE reference values affected results, analyses were also performed with the amount of meat consumed as continuous variable. Altogether, the results were largely comparable but the consideration of reference values allows assessing the practical relevance of the results and improves clarity of interpretation.

## Results

In total, 5757 men and 7158 women aged 18–80 years completed the interview and two 24-h recalls. Participants had a mean age of 48 years and about 30 % had completed 12 or 13 years of school education. A description of the study sample regarding the variables used in the analysis is presented in Table 2.

**Table 2.** Characteristics of the German National Nutrition Survey II (Numbers of participants and percentages)

	Men (44·6 %)		Women (55·4 %)	
Characteristic	*n*	%	*n*	%
Age group (years)	5757		7158	
18–34		20·6		20·1
35–64		59·2		61·3
65–80		20·2		18·6
Education (score)	5757		7158	
Low		27·9		32·7
Middle		29·2		33·2
High		43·0		34·0
Occupational status of the principal earner (score)	5757		7158	
Low		35·4		34·9
Middle		46·7		50·3
High		17·9		14·8
Per capita income	5130		6069	
Low		29·7		35·1
Middle		31·3		29·6
High		39·0		35·3
Country of birth	5744		7142	
Germany		91·6		91·0
Another country		8·4		9·0
Regional structure	5757		7158	
Small cities and rural areas		28·9		26·9
Medium cities and suburbs		35·2		35·3
Large cities		35·9		37·8
Household size	5753		7152	
1 person		14·0		15·1
2 persons		40·7		40·3
3 and more persons		45·3		44·6
BMI	5757		7158	
Underweight		0·6		1·6
Normal weight		34·2		50·8
Preobese		47·1		29·2
Obese		18·1		18·4
Smoking status	5748		7145	
Smoker		23·5		18·2
Occasional smoker		6·0		5·2
Former smoker		29·1		18·6
Non-smoker		41·4		58·0
Subjective health status	5751		7150	
Very good		17·1		16·0
Good		58·3		60·7
Moderate		20·9		20·0
Poor		3·2		2·8
Very poor		0·5		0·5
Sports activities*	3863		4968	
Yes		57·3		*59·9*
No		42·7		*40·1*

* Information obtained from an additional questionnaire in a subsample.

### Meat consumption level

Based on the self-reports checked for consistency, forty-four male (0·8 %) and 138 female (1·9 %) non-meat consumers between 18 and 80 years of age were identified.

Among meat consumers, the mean daily meat consumption was 118 g (Table 1) (total sample with non-meat consumers: 116 g; median daily meat consumption: 99 g). On average, women's meat consumption was at the edge of the maximum recommended amount of 86 g/d. In contrast, men's average meat consumption exceeded this value by 80 %. A higher consumption among men compared with women was observed for all types of meat. When comparing energy-adjusted values, differences between men and women were smaller but still significant, except for meats of special origin (data not shown).

However, the different types of meat constituted a comparable proportion of all meats among men and women: For both sexes, the consumption of meat and meat products accounted for about two-thirds of the total meat consumption while one-third was comprised of sausages. Pork and pork products were by far the most consumed. Meat of unknown or mixed origins – of which about half was minced meat – and poultry were consumed in comparable amounts, while beef, veal, mutton or lamb was consumed in slightly lower amounts.

Considering their individual daily meat consumption, a little less than half (44 %) of women and almost three-quarters (73 %) of men were classified as high meat consumers exceeding the maximum recommended daily intake of 86 g meat per d.

### Sociodemographic characteristics and meat consumption level

**Fig. 1.**
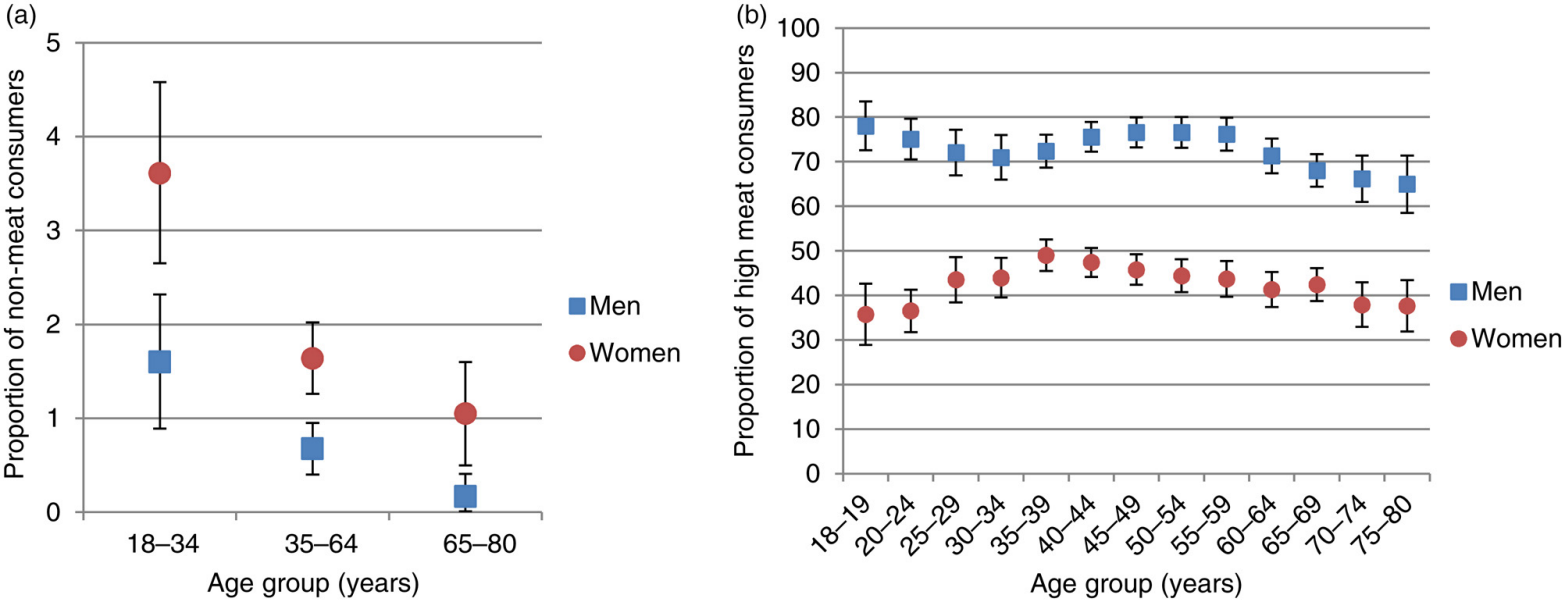
Prevalences (with 95 % confidence intervals) of non-meat consumption (a) and high meat consumption (b) among selected age groups, German National Nutrition Survey II.

Besides the pronounced sex-specific differences in the prevalence of non-meat consumption, low and high meat consumption, some further noteworthy sociodemographic differences were observed. Fig. 1 shows the prevalence of non-meat consumption and high meat consumption in selected age groups, while Table 3 shows the prevalence rates for further sociodemographic groups.

**Table 3.** Prevalences of non-meat consumption, and low and high meat consumption among sociodemographic groups, German National Nutrition Survey II (Percentages and 95 % confidence intervals)

	Men						Women					
			Meat consumers (*n* 5713)						Meat consumers (*n* 7020)			
	Non-meat consumers (*n* 44)		Low meat consumers (*n* 1509)		High meat consumers (*n* 4204)		Non-meat consumers (*n* 138)		Low meat consumers (*n* 3899)		High meat consumers (*n* 3121)	
	%	95 % CI	%	95 % CI	%	95 % CI	%	95 % CI	%	95 % CI	%	95 % CI
												
All participants	0·8	0·6, 1·0	26·2	25·1, 27·4	73·0	71·9, 74·2	1·9	1·6, 2·3	54·5	53·3, 55·6	43·6	42·5, 44·8
Education												
Low	0·1	0·0, 0·4	22·7	20·1, 24·8	77·2	75·0, 79·2	0·9	0·6, 1·4	52·8	50·7, 54·8	46·3	44·3, 48·4
Middle	0·7	0·4, 1·3	22·9	20·9, 25·0	76·4	74·2, 78·4	1·6	1·1, 2·2	52·4	50·4, 54·4	46·0	44·0, 48·0
High	1·2	0·8, 1·7	30·7	28·9, 32·6	68·1	66·2, 69·9	3·2	2·6, 4·0	58·1	56·1, 60·1	38·7	36·7, 40·6
Occupational status of principal earner												
Low	0·7	0·4, 1·2	22·6	20·8, 24·5	76·6	74·7, 78·4	1·3	0·9, 1·9	51·8	49·8, 53·8	46·9	44·9, 48·9
Middle	0·8	0·5, 1·2	28·2	26·5, 30·0	70·9	69·2, 72·7	2·3	1·8, 2·8	55·9	54·3, 57·6	41·8	40·2, 43·1
High	0·7	0·3, 1·4	28·0	25·3, 30·8	71·3	68·5, 74·1	2·2	1·4, 3·2	55·8	52·8, 58·8	42·0	39·0, 45·1
Per capita income												
Low	0·7	0·4, 1·3	23·4	21·3, 25·6	75·9	73·7, 78·0	1·5	1·0, 2·1	51·5	49·4, 53·6	47·0	44·9, 49·2
Middle	0·2	0·1, 0·6	25·8	23·7, 28·0	73·9	71·7, 76·1	1·2	0·7, 1·8	54·8	52·4, 57·1	44·1	41·7, 46·4
High	1·1	0·6, 1·6	28·7	26·7, 30·7	70·3	68·2, 72·3	2·5	1·9, 3·2	57·6	55·4, 59·7	40·0	37·9, 42·1
Country of birth												
Germany	0·8	0·5, 1·0	26·2	25·0, 27·4	73·0	71·8, 74·2	2·0	1·7, 2·4	54·6	53·4, 55·8	43·4	42·2, 44·6
Another country	0·8	0·2, 2·1	25·9	22·0, 30·0	73·3	69·1, 77·2	1·1	0·4, 2·2	53·2	49·2, 57·1	45·7	41·8, 49·7
Regional structure												
Small cities and rural areas	0·7	0·4, 1·3	22·5	20·5, 24·6	76·8	74·6, 78·8	1·5	1·0, 2·1	52·3	50·0, 54·5	46·3	44·0, 48·5
Medium cities and suburbs	0·5	0·2, 0·9	26·6	24·7, 28·6	72·9	70·9, 74·9	1·7	1·2, 2·3	51·7	49·7, 53·6	46·6	44·7, 48·6
Large cities	1·1	0·7, 1·6	28·8	26·9, 30·8	70·1	68·1, 72·1	2·5	1·9, 3·1	58·7	56·8, 60·5	38·9	37·0, 40·7
Household size												
1 person	2·2	1·3, 3·5	28·1	25·0, 31·4	69·7	66·3, 72·8	3·0	2·0, 4·2	60·5	57·5, 63·4	36·6	33·7, 39·5
2 persons	0·4	0·2, 0·8	28·9	27·0, 30·7	70·7	68·8, 72·6	1·6	1·2, 2·2	56·4	54·5, 58·2	42·0	40·2, 43·8
3 and more persons	0·6	0·3, 1·0	23·3	21·7, 25·0	76·1	74·4, 77·7	1·9	1·4, 2·4	50·8	49·0, 52·5	47·4	45·6, 49·1

The prevalence of non-meat consumption was highest among younger adults (18–34 years), in a middle range among middle-aged adults (35–64 years) and lowest among the elderly (65–80 years) for both men and women (Fig. 1(a)). The proportion of non-meat consumers was also higher among more educated persons, among persons with high compared with middle (but not low) per capita income, among persons living in large cities (compared with persons in both other regions combined), and among persons living in single households. Multiple logistic regression models with sociodemographic characteristics as independent variables and non-meat consumption as the dependent variable (Table 4) confirm sex, age, education and household size as independent predictors of non-meat consumption. Separate results for men and women are presented for the sake of completeness, but especially for men the sample size is too small to detect meaningful differences.

**Table 4.** Sociodemographic predictors of non-meat consumption, German National Nutrition Survey II† (Odds ratios and 95 % confidence intervals)

	Non-meat consumption (Ref. meat consumption)					
	Men (*n* 5130) (*R*^2^ 0·12)		Women (*n* 6069) (*R*^2^ 0·06)		Total (*n* 11 199) (*R*^2^ 0·09)	
	OR	95 % CI	OR	95 % CI	OR	95 % CI
Sex						
Men	–	–	–	–	Ref.	
Women	–	–	–	–	2·65*	1·81, 3·89
Age group (years)						
18–34	5·29*	1·13, 24·74	2·51*	1·23, 5·13	3·11*	1·66, 5·85
35–64	2·66	0·59, 11·89	1·33	0·68, 2·60	1·61	0·89, 2·94
65–80	Ref.					
Education						
Low	Ref.					
Middle	9·51*	1·22, 74·21	1·73	0·89, 3·37	2·23*	1·21, 4·10
High	12·58*	1·67, 94·45	3·26*	1·74, 6·09	3·81*	2·14, 6·79
Per capita income						
Low	Ref.					
Middle	0·50	0·15, 1·66	0·83	0·47, 1·47	0·74	0·44, 1·25
High	0·92	0·41, 2·07	1·23	0·74, 2·04	1·12	0·73, 1·72
Regional structure						
Small cities and rural areas	Ref.					
Medium cities and suburbs	0·60	0·23, 1·53	1·04	0·59, 1·86	0·90	0·56, 1·47
Large cities	0·99	0·45, 2·19	1·57	0·93, 2·66	1·39	0·90, 2·15
Household size						
1 person	4·06*	1·67, 9·89	1·86*	1·04, 3·33	2·46*	1·52, 3·98
2 persons	1·33	0·51, 3·48	1·24	0·77, 2·01	1·28	0·83, 1·97
3 and more persons	Ref.					

Ref., reference.

* Significant difference indicated when 95 % CI does not include the value ‘1’.

† OR from multiple logistic regressions with non-meat consumption (Ref. meat consumption) as the dependent variable and sociodemographic factors as independent variables.

The detailed examination of the prevalence of high meat consumption among age groups (Fig. 1(b)) revealed no clear pattern. Among men, high meat consumption was more prevalent among young and middle-aged adults compared with elderly. Among women, the proportion of high meat consumers was highest among middle-aged adults and comparable among young adults and elderly. High meat consumption was also more prevalent among persons with lower socio-economic status (specifically among those with low and middle education, low occupational status of the principal earner and low per capita income), among those living in small cities and rural areas (compared with large cities) and among those living in multi-person households (compared with single- and two-person households) for both sexes (Table 3). In Table 5, the results of separate multiple logistic regression models for men, women and the total sample are displayed with sociodemographic factors as independent variables and high meat consumption as the dependent variable. Male sex could be confirmed as a major predictor of high meat consumption. The model including sex explains about 14 % of the variance in meat consumption level while the sex-specific models can only explain about 3 % of the variance. Lower education and a rather rural setting could be confirmed as independent predictors of high meat consumption among men and women. Age, however, was only significant among males while household size was only significant among females in the adjusted models.

**Table 5.** Sociodemographic predictors of high meat consumption, German National Nutrition Survey II† (Odds ratios and 95 % confidence intervals)

	High meat consumption (Ref. low meat consumption)					
	Men (*n* 5094) (*R*^2^ 0·03)		Women (*n* 5964) (*R*^2^ 0·03)		Total (*n* 11 058) (*R*^2^ 0·14)	
	OR	95 % CI	OR	95 % CI	OR	95 % CI
Sex						
Men	–	–	–	–	3·68*	3·39, 4·00
Women	–	–	–	–	Ref.	
Age group (years)						
18–34	1·39*	1·11, 1·72	1·04	0·86, 1·26	1·20*	1·04, 1·39
35–64	1·53*	1·29, 1·82	1·16	0·99, 1·35	1·33*	1·19, 1·48
65–80	Ref.					
Education						
Low	1·50*	1·26, 1·80	1·32*	1·14, 1·52	1·40*	1·26, 1·57
Middle	1·38*	1·18, 1·62	1·30*	1·14, 1·48	1·34*	1·21, 1·48
High	Ref.					
Occupational position						
Low	1·14	0·92, 1·40	1·13	0·95, 1·35	1·14	1·00, 1·30
Middle	0·97	0·85, 1·21	0·97	0·82, 1·13	0·97	0·86, 1·09
High	Ref.					
Per capita income						
Low	1·02	0·85, 1·21	1·00	0·86, 1·15	1·01	0·90, 1·13
Middle	1·06	0·90, 1·25	0·95	0·82, 1·10	1·00	0·89, 1·11
High	Ref.					
Regional structure						
Small cities and rural areas	1·31*	1·11, 1·54	1·24*	1·08, 1·41	1·26*	1·14, 1·40
Medium cities and suburbs	1·07	0·92, 1·24	1·30*	1·15, 1·47	1·20*	1·10, 1·32
Large cities	Ref.					
Household size						
1 person	Ref.					
2 persons	1·02	0·83, 1·25	1·20*	1·01, 1·42	1·10	0·96, 1·24
3 and more persons	1·19	0·96, 1·47	1·53*	1·27, 1·84	1·35*	1·18, 1·55

Ref., reference.

* Significant difference indicated when 95 % CI does not include the value ‘1’.

† OR from multiple logistic regressions with high meat consumption (Ref. low meat consumption) as the dependent variable and sociodemographic factors as independent variables.

### Meat consumption level and health-related characteristics

Female non-meat consumers were more likely to have a normal weight, do sports and consider their health status as very good compared with female meat consumers (Table 6). For males, similar differences in weight and subjective health were observed in bivariate analyses but did not reach significance in the adjusted logistic regression models.

**Table 6.** Association between level of meat consumption and health-related characteristics, German National Nutrition Survey II† (Odds ratios and 95 % confidence intervals)

	Normal weight (Ref. preobese, obese‡)		Non-smoking/former smoking (Ref. smoking, occasionally smoking)		Very good subjective health (Ref. good, moderate, poor, very poor subjective health)		Sports activities§ (Ref. ‘no’)	
	OR	95 % CI	OR	95 % CI	OR	95 % CI	OR	95 % CI
Non-meat consumption (Ref. meat consumption), unadjusted								
Men	2·94*	1·59, 5·44	1·12	0·57, 2·17	2·79*	1·51, 5·18	1·97	0·87, 4·45
Women	4·18*	2·70, 6·47	1·26	0·83, 1·93	1·81*	1·23, 2·67	1·98*	1·26, 3·10
Non-meat consumption (Ref. meat consumption), adjusted						
Men	1·25	0·60, 2·57	1·43	0·67, 3·04	1·72	0·82, 3·57	1·26	0·53, 3·01
Women	2·55*	1·57, 4·14	1·20	0·74, 1·95	1·58*	1·00, 2·48	1·82*	1·07, 3·10
High meat consumption (Ref. low meat consumption), unadjusted								
Men	0·86*	0·76, 0·97	0·75*	0·66, 0·86	0·93	0·80, 1·09	0·92	0·79, 1·06
Women	0·77*	0·70, 0·84	0·76*	0·68, 0·85	0·96	0·84, 1·09	0·68*	0·60, 0·76
High meat consumption (Ref. low meat consumption), adjusted						
Men	0·89	0·77, 1·03	0·82*	0·70, 0·95	0·96	0·91, 1·14	0·95	0·81, 1·11
Women	0·77*	0·69, 0·86	0·78*	0·69, 0·89	1·05	0·91, 1·21	0·71*	0·62, 0·80

Ref., reference.

* Significant difference indicated when 95 % CI does not include the value ‘1’.

† OR from simple and multiple logistic regressions (adjusted for age, education, occupational position of the principal earner, per capita income, country of birth, regional structure, household size) with non-meat consumption or high meat consumption as the independent variables and health-related characteristics as dependent variables.

‡ Underweight subjects were excluded from the analysis.

§ Information obtained from an additional questionnaire in a subsample.

Among female meat consumers, those with high meat consumption were less likely to have a normal weight, do sports and be a non-smoker. Among male meat consumers, a significant difference in the adjusted models was only observed for smoking status; male high meat consumers were less likely to be non-smokers than low meat consumers.

### Meat consumption level and energy intake and food consumption

There was no significant difference in energy intake between non-meat consumers and meat consumers, although non-meat consumers tended to consume foods of lower energy density (Table 7). They ate more cereals and cereal products, total vegetables (vegetables, vegetable products, mushrooms and pulses) and soya products compared with meat consumers (Tables 8 and 9). Female non-meat consumers additionally ate more fruit and fruit products, nuts and seeds and sauces and spicy ingredients but less of potatoes and potato products, eggs and fats and oils compared with female meat consumers. Since there were no differences in energy intakes between non-meat consumers and meat consumers, energy adjustment alone had no effect on these results. When additionally adjusting for sociodemographic differences, male non-meat consumers ate more bread, potatoes and potato products, total vegetables and soya products and female non-meat consumers ate more cereals and cereal products, total vegetables, fruit and fruit products, nuts and seeds and soya products compared with meat consumers. To summarise, non-meat consumers ate more foods of plant origin and a similar amount of other foods of animal origin compared with meat consumers.

**Table 7.** Association between level of meat consumption and energy intake and energy density, German National Nutrition Survey II† (Regression coefficients (*B*) and 95 % confidence intervals)

	Energy intake (kJ)		Energy intake from meat (kJ)		Energy density (kJ/g)	
	*B*	95 % CI	*B*	95 % CI	*B*	95 % CI
Non-meat consumption (Ref. meat consumption), unadjusted						
Men	132	−836, 1099	–	–	−0·63	−1·38, 0·12
Women	−73	−467, 321	–	–	−0·55*	−0·90, −0·20
Non-meat consumption (Ref. meat consumption), adjusted						
Men	−360	−1405, 685	–	–	−0·86*	−1·66, −0·06
Women	−208	−656, 239	–	–	−0·64*	−1·04, −0·26
High meat consumption (Ref. low meat consumption), unadjusted						
Men	1883*	1698, 2068	1398*	1347, 1448	0·74*	0·59, 0·89
Women	1212*	1106, 1319	911*	890, 933	0·61*	0·51, 0·70
High meat consumption (Ref. low meat consumption), adjusted						
Men	1769*	1576, 1963	1360*	1306, 1414	0·66*	0·51, 0·81
Women	1244*	1129, 1359	915*	891, 938	0·60*	0·50, 0·70

Ref., reference.

* Significant difference indicated when 95 % CI does not include the value ‘0’.

† Regression coefficients from simple and multiple linear regressions (adjusted for age, education, occupational position of the principal earner, per capita income, country of birth, regional structure, household size) with non-meat consumption or high meat consumption as the independent variable and energy intake or energy density as the dependent variables.

**Table 8.** Association between level of meat consumption and consumption of bread, pastries, cereals, potatoes, vegetables, fruit and dairy products, German National Nutrition Survey II† (Regression coefficients (*B*) and 95 % confidence intervals)

	Bread		Pastries		Cereals and cereal products		Potatoes and potato products		Vegetables, vegetable products, mushrooms and pulses		Fruit and fruit products (without juice)		Milk, dairy products and cheese	
	*B*	95 % CI	*B*	95 % CI	*B*	95 % CI	*B*	95 % CI	*B*	95 % CI	*B*	95 % CI	*B*	95 % CI
Non-meat consumers (Ref. meat consumers)														
Men	14·9	−11·1, 41·0	5·1	−17·9, 28·1	41·1*	14·5, 67·8	18·9	−3·3, 41·1	41·1*	10·0, 72·2	43·6	−10·1, 97·4	58·6	−1·4, 118·6
Women	−3·9	−14·3, 6·4	1·1	−9·9, 12·1	32·0*	20·2, 43·8	−11·0*	−21·1, −1·0	59·3*	42·9, 75·7	60·9*	29·8, 92·0	16·0	−13·2, 45·2
Non-meat consumers (Ref. meat consumers), adjusted for energy intake														
Men	13·3	−9·9, 47·4	4·0	−17·4, 25·3	40·7*	14·3, 67·2	18·3	−3·5, 40·2	40·4*	9·7, 71·2	42·8	−10·6, 96·2	56·5	−1·5, 114·6
Women	−3·2	−12·5, 6·2	2·0	−8·1, 12·0	32·4*	20·7, 44·0	−10·8*	−20·7, −0·8	59·8*	43·6, 76·0	61·8*	31·1, 92·5	17·7	−10·2, 45·5
Non-meat consumers (Ref. meat consumers), adjusted for energy intake and sociodemographic variables														
Men	30·5*	5·1, 55·9	7·0	−16·7, 30·6	20·5	−8·3, 49·4	33·8*	10·2, 57·4	43·8*	9·8, 77·7	54·1	−4·1, 112·4	55·5	−8·3, 119·3
Women	3·7	−7·1, 14·4	2·6	−8·9, 14·1	21·0*	7·9, 34·1	−9·7	−21·0, 1·6	61·6*	43·1, 80·2	69·6*	34·8, 104·3	9·0	−23·1, 41·0
High meat consumption (Ref. low meat consumption)														
Men	16·1*	11·0, 21·2	2·1	−2·4, 6·7	−6·2*	−11·4, −0·9	21·3*	17·0, 25·7	2·4	−3·7, 8·6	−29·6*	−40·2, −19·0	−20·1*	−32·6, −8·9
Women	10·6*	7·7, 13·4	5·7*	2·6, 8·8	1·1	−2·1, 4·4	12·9*	10·2, 15·7	1·6	−2·9, 6·1	−33·7*	−42·2, −25·1	−24·9*	−33·0, −16·7
High meat consumption (Ref. low meat consumption), adjusted for energy intake														
Men	−7·2*	−11·9, −2·4	−15·3*	−19·7, −10·9	−13·3*	−18·7, −7·9	14·6*	10·1, 19·0	−7·5*	−13·7, −1·2	−44·3*	−55·2, −33·4	−53·5*	−65·4, −41·7
Women	−2·7*	−5·4, −0·0	−9·1*	−12·0, −6·2	−5·4*	−8·8, −2·1	8·8*	6·0, 11·7	−6·2*	−10·8, −1·6	−51·6*	−60·3, −42·9	−56·0*	−63·9, −48·1
High meat consumption (Ref. low meat consumption), adjusted for energy intake and sociodemographic variables														
Men	−7·5*	−12·5, −2·5	−15·2*	−19·9, −10·6	−15·8*	−21·4, −10·1	15·5*	10·9, 20·1	−5·4	−12·1, 1·2	−40·9*	−52·3, −29·4	−49·4*	−61·9, −36·8
Women	−3·5*	−6·5, −0·6	−10·3*	−13·4, −7·1	−5·2*	−8·8, −1·6	8·6*	5·5, 11·7	−6·6*	−11·6, −1·6	−49·3*	−58·7, −39·9	−52·6*	−61·3, −43·8

Ref., reference.

* Significant difference indicated when 95 % CI does not include the value ‘0’.

† Regression coefficients from simple and multiple linear regressions (adjusted for energy intake only and adjusted for energy intake, age, education, occupational position of the principal earner, per capita income, country of birth, regional structure, household size) with non-meat consumption or high meat consumption as the independent variable and food consumption as dependent variables.

**Table 9.** Association between level of meat consumption and consumption of fish, eggs, fats and oils, nuts and seeds, soups, sauces and spicy ingredients, confectionery and soya products, German National Nutrition Survey II† (Regression coefficients (*B*) and 95 % confidence intervals)

	Fish, fish products and seafood		Eggs		Fats and oils		Nuts and seeds		Soups		Sauces and spicy ingredients		Confectionery		Soya products	
	*B*	95 % CI	*B*	95 % CI	*B*	95 % CI	*B*	95 % CI	*B*	95 % CI	*B*	95 % CI	*B*	95 % CI	*B*	95 % CI
Non-meat consumers (Ref. meat consumers)																
Men	1·8	−11·7, 15·4	2·9	−4·2, 10·0	−1·1	−8·2, 5·9	2·7	−1·0, 6·5	−22·4	−57·1, 12·3	6·7	−2·6, 16·1	8·5	−8·8, 25·7	58·1*	51·5, 64·6
Women	−4·6	−10·6, 1·4	−4·3*	−7·7, −0·8	−2·6*	−5·1, –0·1	3·9*	2·1, 5·6	8·1	−8·2, 24·3	5·2*	0·3, 10·0	−7·0	−15·7, 1·6	25·0*	20·4, 29·6
Non-meat consumers (Ref. meat consumers), adjusted for energy intake																
Men	1·7	−11·8, 15·3	2·8	−4·2, 9·8	−1·6	−7·8, 4·6	2·6	−1·1, 6·4	−22·5	−57·2, 12·2	6·5	−2·7, 15·6	7·5	−8·2, 23·3	58·0*	51·5, 64·6
Women	−4·5	−10·5, 1·4	−4·2*	−7·6, −0·8	−2·4*	−4·6, −0·2	3·9*	2·2, 5·7	8·3	−7·9, 24·5	5·3*	0·6, 10·0	−6·4	−14·4, 1·6	25·0*	20·4, 29·6
Non−meat consumers (Ref. meat consumers), adjusted for energy intake and sociodemographic variables																
Men	2·4	−12·7, 17·6	2·9	−5·0, 10·8	0·7	−5·9, 7·4	4·0	−0·2, 8·2	−18·6	−57·4, 20·3	−1·2	−11·2, 8·8	−5·6	−22·9, 11·7	47·3*	40·5, 54·2
Women	−4·1	−11·0, 2·8	−3·6	−7·6, 0·4	−0·5	−3·0, 2·0	2·4*	0·4, 4·4	15·2	−3·5, 34·0	4·1	−1·3, 9·5	−8·0	−17·2, 1·2	19·8*	15·2, 24·4
High meat consumption (Ref. low meat consumption)																
Men	−16·2*	−18·8, −13·5	−0·6	−2·0, 0·8	5·4*	4·0, 6·7	−0·3	−1·1, 0·4	−13·3*	−20·1, −6·4	9·2*	7·3, 11·0	3·7*	0·3, 7·1	−1·0*	−2·0, −0·0
Women	−8·6*	−10·2, −6·9	0·6	−0·4, 1·5	3·5*	2·8, 4·2	−0·6*	−1·0, −0·1	−10·7*	−15·2, −6·2	7·6*	6·3, 8·9	4·3*	1·9, 6·8	−2·2*	−3·4, −1·1
High meat consumption (Ref. low meat consumption), adjusted for energy intake																
Men	−19·0*	−21·7, −16·2	−2·6*	−4·0, −1·1	−1·4*	−2·6, −0·1	−1·5*	−2·3, −0·8	−15·4*	−22·6, −8·3	5·9*	4·0, 7·8	−10·8*	−14·0, −7·6	−1·3*	−2·3, −0·2
Women	−10·6*	−12·3, −8·9	−0·7	−1·7, 0·2	0·1	−0·5, 0·8	−1·4*	−1·9, −0·9	−14·7*	−19·4, −10·1	5·2*	3·9, 6·6	−6·9*	−9·2, −4·6	−2·4*	−3·6, −1·3
High meat consumption (Ref. low meat consumption), adjusted for energy intake and sociodemographic variables																
Men	−18·9*	−21·9, −16·0	−2·9*	−4·4, −1·3	−1·0	−2·3, 0·3	−1·4*	−2·2, −0·6	−15·2*	−22·9, −7·6	5·4*	3·4, 7·3	−10·2*	−13·6, −6·8	−0·7	−1·8, 0·4
Women	−10·5*	−12·4, −8·6	−0·9	−2·0, 0·2	0·2	−0·5, 0·9	−1·5*	−2·1, −1·0	−14·7*	−19·8, −9·5	5·4*	3·9, 6·9	−6·6*	−9·1, −4·0	−2·7*	–3·9, −1·6

Ref., reference.

* Significant difference indicated when 95 % CI does not include the value ‘0’.

† Regression coefficients from simple and multiple linear regressions (adjusted for energy intake only and adjusted for energy intake, age, education, occupational position of the principal earner, per capita income, country of birth, regional structure, household size) with non-meat consumption or high meat consumption as the independent variable and food consumption as dependent variables.

Among meat consumers, substantial differences in energy intakes were observed: male and female high meat consumers' energy intake was 1883 and 1212 kJ higher than that of low meat consumers, respectively (Table 7). Solely from meat, male and female high meat consumers had a higher energy intake of 1398 and 911 kJ compared with low meat consumers, respectively. High meat consumers also consumed foods of higher energy density.

High meat consumers of both sexes ate more bread, potatoes and potato products, fats and oils, sauces and spicy ingredients and confectionery but less fruit and fruit products, milk, dairy products and cheese, fish, fish products and seafood, soups and soya products than low meat consumers (Tables 8 and 9). Since both groups of meat consumers differed in their energy intake, energy adjustment seriously affected these results. When taking the difference in energy intake into account, high meat consumers ate more potatoes and potato products, and sauces and spicy ingredients but less of most other food groups compared with low meat consumers. An additional adjustment for the sociodemographic differences had only minor effects on these results.

## Discussion

This study described meat consumption and analysed the sociodemographic and health-related characteristics as well as the consumption behaviour of non-meat consumers, and low and high meat consumers in Germany. It revealed several key findings:

1. In Germany, meat is consumed in large amounts: More than a half of the population consumed more meat than recommended.
2. Gender aspects shape meat consumption behaviour: Among men, a lower proportion of non-meat consumers and a higher proportion of high meat consumers were observed compared with women.
3. Age and education but also living environments also shape meat consumption behaviour: Non-meat consumption is more frequent among young and better-educated persons and among those living in single households, while high meat consumption is more frequent among young and middle-aged and lower-educated persons as well as among those living in small cities and rural areas and in larger households.
4. Meat consumption behaviour is related to health-related lifestyles: While non-meat consumers showed equal or more preferable health characteristics compared with meat consumers, high meat consumers showed equal or less preferable health characteristics compared with low meat consumers.
5. Non-meat consumers do not substitute meat with other animal-based foods: They had an equal energy intake but ate more plant-based foods and did not eat more fish/fish products/seafood, milk/dairy products/cheese or eggs compared with meat consumers.
6. High meat consumers have high energy intakes and higher intakes of the traditional meal pattern components meat, potatoes and sauce: Their energy intake, especially their energy intake from meat, was higher compared with low meat consumers. When adjusting for energy intake, they ate more potatoes/potato products and sauces/spicy ingredients compared with low meat consumers and less of most other food groups.

### Meat consumption level

In the past 20 years, different results regarding the prevalence of vegetarian diets in Germany have been published ranging from 2 to 10 %^(^^21^^,^^22^^)^, with data from the NVS II showing the lowest prevalence. In the NVS II, trained interviewers inquired about particular types of vegetarian diets (vegan, lacto-vegetarian, ovo-vegetarian, ovo-lacto-vegetarian or pesco-ovo-lacto-vegetarian) and provided the corresponding definitions when necessary. Additionally, the classification of a vegetarian diet was not enlarged by attributes like ‘usually’, ‘largely’ or ‘predominantly’. This strict definition probably explains the low prevalence compared with other studies. Additionally, in the German National Nutrition Monitoring, a longitudinal study based on the NVS II, an increase in the prevalence of vegetarian diets was observed^(^^23^^)^. Therefore, changes over time might also explain part of the differences. Compared with previous NVS II results, the present analysis resulted in an even lower prevalence of non-meat consumers as additional consistency checks were performed with consumption data. Although the prevalences vary widely due to different assessment methods and definitions^(^^21^^,^^22^^,^^24^^)^ and vegetarian diets seem to gain popularity, the numbers indicate that only a minority of the German population follows a strict meat-free diet.

Instead, many Germans consume more meat than recommended and an adjustment of meat consumption to the dietary guidelines would require a behavioural change in more than half of the population. The literature on barriers and opportunities for meat reduction indicates that such a drastic change is unlikely to occur unless social–cultural transformation processes are initiated as well^(^^25^^,^^26^^)^.

### Sociodemographic characteristics and meat consumption level

The results confirm a large number of different studies linking female sex, younger age and better education to a non-meat/vegetarian diet^(^^21^^,^^22^^,^^27^^,^^28^^)^. The higher proportion of female non-meat consumers/vegetarians is usually explained by sociocultural aspects. According to the literature, meat is perceived as man's food with its symbolic associations with strength and power. Women, on the other hand, often show a lower preference for meat as they have stronger ethical concerns regarding animal welfare, are more interested in nutritional topics, and show a higher health and figure awareness^(^^24^^,^^26^^,^^29^^)^. For the higher proportion of non-meat consumers/vegetarians in younger and more-educated groups, different explanations could apply (e.g. higher interest in new nutritional topics, openness to trends, social positioning purposes (see below), higher environmental awareness). Previous literature also reports a higher proportion of vegetarians among urban residents^(^^21^^,^^28^^)^, probably for similar reasons. However, similar differences in the proportion of non-meat consumers could not be confirmed here. That a non-meat/vegetarian diet is more often observed among persons in single households – or as previously mentioned among persons living in smaller households^(^^28^^)^ and among singles^(^^27^^)^ – might indicate that following such a restrictive diet is easier without coordination and negotiation processes with other household members.

Higher consumption of meat and its subtypes, unadjusted and adjusted for energy intake, among men has been comprehensively reported in the literature^(^^30^^,^^31^^)^. Therefore, the higher meat consumption among men cannot be explained by men's generally higher energy requirements alone. Instead, as previously stated, different meat consumption patterns among sexes are usually explained by sociocultural aspects^(^^24^^,^^26^^,^^29^^)^. Traditional sex-specific role models and corresponding expectations still shape food consumption behaviour in general and meat consumption in particular. To this day meat consumption is shaped by traditional sex-specific role models and corresponding expectations. An inverse relationship between education and meat consumption has also been frequently reported^(^^30^^–^^32^^)^. This might be due to greater health orientation and a higher awareness of the adverse effects of meat consumption among better-educated individuals. Limited consumption of meat might also function as a means of social distinction. Today, meat is obtainable (almost) everywhere at relatively low prices and it seems to have lost its attribute as a status symbol. Therefore, reducing meat consumption or entirely abstaining from meat may appear morally superior due to the adverse ethical, ecological and health effects of (high) meat consumption^(^^29^^)^. According to Schlegel-Matthies^(^^29^^)^, such processes of social positioning might also explain differences in meat consumption between urban and rural residents, as observed here. Besides, lower meat consumption among urban residents might be explained by their openness towards new trends as well as a higher availability and variety of vegetarian options in cities.

Gossard & York^(^^32^^)^ and Linseisen *et al*.^(^^31^^)^ report that the amount of consumed meat decreases with increasing age. In this sample, a similar trend could be observed, and it seems reasonable that older persons consume less meat as a result of lower energy needs and higher health awareness. However, our results showed that a linear model is not optimally suited to describe the relationship between age and proportion of high meat consumers. Therefore, the association between age and meat consumption might warrant further investigation. This might also apply to household size. Our results suggest that females might adopt higher meat consumption of other household members, but meat consumption was seldom analysed according to household size or family status before, which makes this a hypothetical assumption.

To conclude, sociodemographic groups show differences in their meat consumption level and there is good evidence that initiatives and campaigns aiming to reduce meat consumption to the recommended level should primarily address men as well as young and middle-aged and persons with lower education. However, it must be noted that differences except for sex are not very large and that sociodemographic characteristics only explain a small part of the variation in meat consumption level. Differences in meat consumption might rather be explained by different attitudes. Another analysis (in preparation) should provide further information in this regard.

### Meat consumption level and health-related characteristics

More preferable health characteristics – in terms of BMI, sports activities and smoking status – among vegetarians compared with meat consumers were previously reported^(^^21^^,^^27^^,^^33^^)^. The results suggest that a non-meat/vegetarian diet can be considered as part of an overall health-conscious lifestyle. This is not surprising since personal health is one of the main motivations to adopt a vegetarian diet^(^^24^^)^.

Also consistent with our findings, associations between higher meat consumption and less favourable health-related characteristics were previously described^(^^30^^,^^31^^)^. Therefore, high meat consumption might be embedded in an overall less healthy lifestyle. This suggests that the reduction of high meat consumption to a healthy and sustainable level should be addressed as one of several health behaviours in complex health promotion programmes^(^^34^^)^.

### Meat consumption level and energy intake and food consumption

Our results indicate that non-meat consumers do not substitute meat with fish, eggs or milk products but rather with soya products and other foods of plant origin. Relatively large amounts of cereals, vegetables, fruit, nuts and pulses are described as common for a vegetarian diet^(^^33^^)^. However, previous results reveal no clear answer to the question whether meat is substituted with other animal products in vegetarian diets. For example, Bedford & Barr^(^^27^^)^ found a higher consumption of milk products among male vegetarians while Mensink *et al*.^(^^21^^)^ observed a higher consumption of milk products but a lower consumption of eggs among female vegetarians compared with meat consumers. In contrast, Haddad & Tanzman^(^^35^^)^ found no differences in milk product consumption between vegetarians and non-vegetarians. Nonetheless, the results presented here comply with findings that non-meat consumers/vegetarians to a larger extent adhere to dietary recommendations^(^^35^^)^, which reflects their more health-conscious lifestyle.

Similar to our results, associations between higher levels of meat consumption and higher energy intake have been reported previously^(^^31^^,^^36^^)^. The results further indicate that high meat consumers might more strongly adhere to the classical meal structure of meat (as the centrepiece) with potatoes and gravy. Their diet seems to be less varied compared with those of participants with lower meat consumption. According to de Boer *et al*.^(^^37^^)^ there are different, complementary change strategies to reduce meat consumption in current high-meat-eating societies: Promoting smaller portions of meat in a meal, either in favour of meat raised in a more sustainable manner (‘less but better') or in favour of more vegetable protein (‘less and more varied’), or promoting meatless days (‘veggie days’). Our results suggest that it might be easier for current high meat consumers to reduce meat portions in the preferred traditional meal structure than to adopt new vegetarian meals. In any case, meat-reduced or vegetarian meals need to be highly appealing, easy to prepare and simple to adopt into current habits to be accepted as a genuine alternative.

### Strengths and limitations

This is the first study using the nationally representative data of the NVS II to compare non-meat consumers and meat consumers as well as low meat consumers (meeting the dietary recommendation of the German Nutrition Society to eat no more than 300 to 600 g meat per week) and high meat consumers (eating more meat than recommended). It adds further evidence to previous studies showing that non-meat/vegetarian and low-meat diets are more frequent among women and better-educated persons and that following such a diet seems to fit into an overall healthier lifestyle.

However, the present study has several limitations. It was based on data collected about 10 years ago. In this time span, many trend messages about vegetarian and vegan lifestyles were disseminated and those diets and lifestyles received high media attention. Thus, it is possible that changes have occurred in the prevalence of non-meat diets as well as the factors associated with them. It is also important to note that we analysed non-meat consumers who may include fish in their diet instead of vegetarians. This could have affected the results, which might have been clearer if the focus had been on persons who abstain from eating meat and fish. Moreover, despite the large overall sample size, the number of non-meat consumers was very low, especially for sex-specific analyses. Given the high scientific interest in non-meat consumers, this paper aimed to use the given data to provide detailed and complete information on this group. However, analyses based on this small number warrant serious caution and should be understood as exploratory. Since the data came from a cross-sectional survey, only associations can be reported and interpretations about causality are not permitted.

With regard to the amount of meat consumed, the large time span since data collection is assumed to have no substantial effect on the results. The German National Nutrition Monitoring showed that meat consumption was stable between 2006 and 2014, even among subgroups of the population^(^^38^^)^. The maximum amount of meat consumption recommended by the German Nutrition Society was used as a single cut-off point to divide the meat consumers sample into low and high meat consumers, based on their consumption behaviour on two recall days. This approach might produce misleading results for those individuals whose meat consumption was close to the recommended amount. However, analyses were also performed with the amount of meat consumed as a continuous variable. Since results were largely comparable, the classification according to the reference values was presented for better interpretability.

Despite these limitations, this study can add further valuable insights on (non-)meat consumption in Germany.

### Conclusion

Meat consumption in Germany is above the recommended level. In particular, sex differences shape meat consumption behaviour: men are less likely to abstain from meat and more likely to consume meat in exaggerated amounts. Meat consumption behaviour is further influenced by age, education, household size and rural/urban living environments. Health behaviours and food consumption patterns differ by meat consumption behaviour. Explanations for the findings and implications for a reduction of meat consumption were discussed.

This paper provides further insights to the issue of non-meat consumption and high meat consumption. However, future research also needs to consider attitudes towards meat and social–cultural aspects of meat consumption to gain a better understanding of meat consumption behaviour and ways to reduce it to the recommended level.
